# Changes in Initial Opioid Prescribing Practices After the 2016 Release of the CDC Guideline for Prescribing Opioids for Chronic Pain

**DOI:** 10.1001/jamanetworkopen.2021.16860

**Published:** 2021-07-13

**Authors:** Jason E. Goldstick, Gery P. Guy, Jan L. Losby, Grant Baldwin, Matthew Myers, Amy S. B. Bohnert

**Affiliations:** 1 Injury Prevention Center, University of Michigan, Ann Arbor; 2 Department of Emergency Medicine, University of Michigan, Ann Arbor; 3 Centers for Disease Control and Prevention, Atlanta, Georgia; 4 Department of Anesthesiology, University of Michigan, Ann Arbor; 5 Department of Psychiatry, University of Michigan, Ann Arbor

## Abstract

**Importance:**

The Centers for Disease Control and Prevention (CDC) released the “Guideline For Prescribing Opioids For Chronic Pain” (hereafter, *CDC guideline*) in 2016, but its association with prescribing practices for patients who are opioid naive is unknown.

**Objective:**

To estimate changes in initial prescribing rates, duration, and dosage practices to patients who are opioid naive after the release of the CDC guideline.

**Design, Setting, and Participants:**

This cohort study used 6 sequential cohorts to estimate preguideline trends in prescribing among patients who were opioid naive, project that trend forward, and compare it with postguideline prescribing practices. Participants included commercially insured adults without current cancer or hospice care diagnoses and with no past-year opioid claims in the US from 2011 to 2017. All adjusted models were controlled for patient demographics and state-fixed effects. Data were analyzed from January 2020 to May 2021.

**Exposures:**

The release of the CDC guideline.

**Main Outcomes and Measures:**

Indicators of any opioid prescription fills during a 9-month period, the number of days’ supply of the initial prescription, and the binary indicator of whether the initial prescription was for 50 or more morphine milligram equivalents (MMEs) per day.

**Results:**

There were 12 870 612 eligible unique patients across cohorts (mean [SD] age in 2016, 51.2 [18.7] years; 6 553 458 [50.9%] women); and the mean (SD) age of the cohorts increased annually, from 48.7 (17.9) years in the April 2011 to December 2012 cohort to 51.9 (19.2) years in the April 2016 to December 2017 cohort. The postguideline prescribing prevalence was 532 962 of 5 834 088 individuals (9.1%), which exceeded that projected from the preguideline trend, estimated at 9.0% (95% CI, 9.0%-9.1%). Among patients receiving prescriptions during follow-up, adjusted mean days’ supply was 4.7% (95% CI, 4.3%-5.1%) lower in the first year after release of the guideline and 9.8% (95% CI, 9.3%-10.3%) lower in the second year after release, compared with the expected rate from the preguideline trend. The adjusted odds of receiving a high-dose (ie, ≥50 MME/d) initial prescription were lower in the first year (odds ratio, 0.97; 95% CI, 0.96-0.98) and in the second year (odds ratio, 0.94; 95% CI, 0.93-0.96) after the release of the CDC guideline compared with the odds expected from the preguideline trend.

**Conclusions and Relevance:**

This cohort study found that patients who were opioid naive continued to initiate opioid therapy after the release of opioid prescribing guidelines by the CDC, but trends in prescribing duration reversed and decreased, after increasing in each of 4 preguideline cohorts examined. High-dose prescribing rates were already decreasing, but those trends accelerated after the CDC guideline release. These results suggest that nonmandatory, evidence-based guidelines from trusted sources were associated with prescribing practices. Guideline-concordant care has potential to improve pain management and reduce opioid-related harms.

## Key Points

**Question:**

Did prescribing practices applied to commercially insured patients who were opioid naive change after the 2016 release of the Centers for Disease Control and Prevention (CDC) “Guideline for Prescribing Opioids for Chronic Pain”?

**Findings:**

This cohort study included 12 870 612 patients with commercial claims data to examine whether trends in initial prescribing to patients who were opioid naive departed from preguideline trends after the CDC guideline release. Both initial prescribing duration and dosage were significantly lower after the CDC guideline release than would be expected by extrapolating the preguideline trend.

**Meaning:**

These findings suggest that nonmandatory, evidence-based guidelines from trusted sources may affect clinician prescribing behavior.

## Teaser

This cohort examines trends in opioid prescribing for patients who were opioid naive before and after the release of prescribing guidelines by the Centers for Disease Control and Prevention (CDC).

## Introduction

Opioid prescribing began to increase in the US in the 1990s, continued to increase thereafter, and peaked in 2010,^1,2,3^ resulting in millions of people using opioids for pain management.^4^ Although the public health crisis of opioid overdose has evolved over time, with use of heroin and other synthetic opioids (eg, fentanyl)increasing in relative importance in the past decade, prescription opioid overdose death rates have remained high.^5,6^ In addition, individuals receiving prescription opioids are at increased risk of developing opioid use disorder,^7^ with approximately 4 in 5 people who initiate heroin use having previously misused prescription opioids,^8^ and many patients presenting with heroin overdose have a history of having received an opioid prescription.^9^ Given these facts and the lack of evidence-based guidelines to encourage opioid stewardship, the Centers for Disease Control and Prevention (CDC) released the “Guideline for Prescribing Opioids for Chronic Pain”^10^ in 2016 (hereafter, *CDC guideline*).

The voluntary CDC guideline provides evidence-based recommendations to encourage clinicians in primary care settings to use caution when prescribing opioids for adults with chronic, noncancer pain. The purpose of the guideline is to (1) improve patient-physician communication about the risks and benefits of opioid therapy, (2) enhance safety and effectiveness of pain treatment, and (3) reduce risks of long-term opioid use. When opioids are prescribed, the CDC guideline provides recommendations on dosage and duration, including when opioids are used for acute pain prescribing for a period similar to the expected duration of severe pain (in which 3 days or less is often sufficient and more than 7 days is rarely needed) and the lowest daily dosage at which pain is reduced should be used (with special care taken surrounding dosages of 50 morphine milligram equivalents [MMEs] or more per day). How these recommendations corresponded to changes in prescribing for patients who are opioid naive, beyond just those affected by chronic pain, remains an open question.

Prior analyses of prescribing changes following the release of the CDC guideline are limited to 2 key publications. One 2018 study by Bohnert et al^11^ estimated changes in prescription dispensing using retail pharmacy data and found an acceleration of decreasing trends in overall prescribing and high-dose prescribing. A 2019 study by Fenton et al^12^ focused on prescribing changes among individuals with long-term opioid prescriptions and found an increase in tapering and rates of rapid tapering in 2016 to 2017, coinciding with the CDC guideline release. To our knowledge, none of the prior evaluations have studied how initial prescribing among patients who are opioid naive changed after the guideline release, which is the purpose of this study.

In this cohort study, we used commercial insurance claims data to examine rates of opioid initiation, initial prescribing duration, and initial prescribing dose among patients who were opioid naive from 2011 to 2017. The purpose of this study was to examine trends in prescribing rates, dosage, and duration, and the degree to which actual rates of these outcomes differed from the projected rates, had the pre–CDC guideline trends continued. We hypothesize that rates of initiating prescriptions, and the duration and dosage of those initial prescriptions, decreased after the CDC guideline release, beyond what would be expected from the preexisting trends.

## Methods

The University of Michigan institutional review board determined the study to be exempt from approval and informed consent and not regulated because the study was not considered human participants research because the data do not include identifiers. This study is reported following all applicable Strengthening the Reporting of Observational Studies in Epidemiology (STROBE) reporting guidelines.

### Data Description

The Optum deidentifed Clinformatics Data Mart Database contains enrollment records and all claims (medical and pharmacy) from a large national commercial insurer in the United States, as well as claims covered by Medicare Advantage by the same insurer. Data used for this study span from April 2011 through December 2017 and include beneficiaries from all 50 states, Puerto Rico, and the District of Columbia. In addition to all claims, patient demographics (ie, age, sex, and race/ethnicity) are available.

### Cohort Definitions

To explore changes in prescribing to adults who were opioid naive after the CDC guideline release, we constructed sequential cohorts of individuals aged 18 years and older who received no opioid fills during a 12-month baseline period, and also had data available during a 9-month follow-up period (eFigure 1 in the Supplement). To guard against potential seasonal effects, each cohort’s baseline began in April, and follow-up begins the following April and continued through the end of the year. The 9-month follow-up period was chosen specifically because the CDC guideline was released in March 2016, and a 9-month follow-up period takes the observation window through the end of the year. To establish the preguideline trend, we constructed 4 preguideline groups with follow-up periods from 2012 to 2015. The 2 postguideline cohorts were constructed analogously, with follow-up periods ending at the end of 2016 and 2017. We chose an annual time-step rather than monthly or quarterly owing to significant seasonality in disenrollment rates (eFigure 2 in the Supplement). Specifically, there is a spike in disenrollment each January, which would create heterogeneity across cohorts based on months or quarters; to eliminate this confounder, we ensured that the same month windows were included in every cohort through an annual time-step.

Within each cohort, eligible adults were continuously enrolled during the entire 21-month period and had no opioid fills during the 12-month baseline period. We excluded anyone with cancer or palliative care claims (eTable 1 in the Supplement) during their continuous enrollment period. The proportion of people excluded for cancer diagnosis or receipt of palliative care ranged from 578 312 of 6 412 400 individuals (9.0%) (2016-2017 cohort) to 577 801 of 5 377 611 individuals (10.8%) in the (2014-2015 cohort). Individuals were included in all cohorts in which they met eligibility criteria.

### Quantifying Opioid Prescribing

We quantified opioid dosage using MMEs, calculated by multiplying prescription strength, quantity supplied, and MME conversation factor. Prescription strength, duration, and quantity are each identifiable from the health record data, and the conversation factor was obtained from the CDC’s conversion reference table.^13^ We calculated prescription duration as the number of days covered by the prescription. When there were concurrent prescriptions, we summed MME totals across all prescriptions, and the number of days’ supply is defined as the number of days covered by at least 1 prescription. Opioid prescriptions without MME conversion rates or with missing or incomplete prescribing data were omitted from analysis; this affected 47 537 of 87 365 170 prescriptions (0.05%). Individuals whose only opioid prescription was dropped for this reason were omitted from analyses to minimize misclassification in the opioid naive criteria.

### Outcomes

We used a binary indicator of receiving any opioid prescriptions during follow-up. Among those with a prescription during follow-up, we examined 3 main outcomes: (1) the duration of the initial prescription (in days), (2) the dosage of the initial prescription (in MME per day), and (3) the binary indicator of whether the initial prescription was high dose (ie, ≥50 MME/d).

### Covariates

Our covariates were limited to basic patient characteristics, including age, sex, race/ethnicity, insurance provider (commercial or Medicare Advantage), and residence state. Race/ethnicity is imputed based on an internal algorithm used by Clinformatics, which was modified in recent years resulting in a higher proportion missing. Race/ethnicity was included as a covariate to examine whether there were differences in tendency to receive an opioid prescription by race/ethnicity.

### Statistical Analysis

We began with basic demographic descriptions of each cohort. In service to the primary analytic goal, our first set of analyses compared new prescription rates across cohorts. Among individuals who received new prescriptions, we compared number of days supply and dosages across cohorts, as well as whether the initial dosage during follow-up exceeded 50 MME/d. These comparisons are focused on whether the levels observed during the postguideline period were beyond what would have been expected by the preexisting secular trend. To construct that comparison, we fit a trend to the rates seen in the 4 preguideline groups, and extrapolated 2 years forward to project the postguideline rate to determine what would be expected from a continuation of the preguideline trend. In all cases, a linear trend was sufficient to capture and extrapolate the preguideline trend. We calculated prediction intervals using generalized linear models (eg, logistic regression for binary outcomes) for the postguideline values based on a model fit only to the preguideline trend and tested whether the observed postguideline values differed from those projected by the preexisting trend. We also conducted exploratory analyses within demographic groups. All analyses were conducted using R statistical software version 3.6.2 (R Project for Statistical Computing), and all hypothesis testing was 2-tailed, with statistical significance set at *P* < .05. Data were analyzed from January 2020 to May 2021.

To derive adjusted estimates of the association of the guideline with initial prescription duration during follow-up and the indicator of high-dose prescribing during follow-up, we used overdispersed Poisson generalized linear models for the prescribing duration, and binary logistic generalized linear models for the high-dose prescribing indicator. The unit of analysis in those models is the individual, and only individuals who received a prescription during the follow-up period were included. All models included state fixed and a linear time slope (time: 1-6 for the 6 cohorts), patient-level demographic variables, and insurance type (commercial or Medicare). The primary independent variables were categorical variables for the first and second year after release of the CDC guideline, which quantify the departure from the trend that began with the preguideline rates.

## Results

### Study Population

Across all 6 cohorts there were 12 870 612 unique individuals (mean [SD] age in 2016, 51.2 [18.7] years; 6 553 458 [50.9%] women), and the size of each individual cohort ranged from 4 475 718 individuals in the 2013 to 2014 cohort to 5 832 088 individuals in the 2016 to 2017 cohort. Descriptive summaries of each cohort are shown in Table 1. Every cohort had a approximately even proportions of men and women, included approximately two-thirds White participants (eg, 3 129 761 White individuals [66.5%] in 2012-2013), and were primarily covered with commercial insurance (eg, 3 664 178 individuals [77.9%] in 2012-2013). The percentage missing in the race variable was approximately 9.5% across all cohorts and increased from approximately 9.0% in the first 5 cohorts to 12.0% in the 6th cohort. The mean age of each cohort increased annually, from 48.7 (17.9) years in the 2011 to 2012 cohort to 51.9 (19.2) years in the 2016 to 2017 cohort, as did the percentage of individuals with Medicare Advantage insurance. More detailed analyses of changes over time in the cohorts, stratified by insurer type, are shown in eTables 2-5 in the Supplement. eTable 6 in the Supplement shows demographics of commercially insured individuals from 2012 to 2017 derived from NHIS^14^; the age and sex distribution were broadly consistent with our study population, although the non-Medicare group in our data had a mean age that was a few years older, but the race/ethnicity distribution was difficult to compare, given the large amount of missing race/ethnicity data in the database.

**Table 1.** Description of the 6 Cohorts With No Opioid Fills During the 12-Month Baseline Periods

Cohort^a^	Date range	Total, No.	No. (%)			Age, mean (SD), y
			Women	White race/ethnicity	Commercial insurance^b^	
Preguideline						
1	Apr 2011-Dec 2012	4 706 093	2 391 450 (50.8)	3 129 761 (66.5)	3 664 178 (77.9)	48.7 (17.9)
2	Apr 2012-Dec 2013	4 869 812	2 472 468 (50.8)	3 227 276 (66.3)	3 668 441 (75.3)	49.4 (18.2)
3	Apr 2013-Dec 2014	4 475 718	2 244 397 (50.1)	2 948 098 (65.9)	3 297 627 (73.7)	49.7 (18.5)
4	Apr 2014-Dec 2015	4 799 810	2 422 811 (50.5)	3 125 164 (65.1)	3 484 259 72.6)	50.1 (18.7)
Postguideline						
1	Apr 2015-Dec 2016	5 234 958	2 668 359 (51.0)	3 400 539 (65.0)	3 677 704 (70.3)	51.0 (19.0)
2	Apr 2016-Dec 2017	5 834 088	2 973 689 (51.0)	3 660 149 (62.7)	3 929 201 (67.3)	51.9 (19.2)

^a^ Preguideline indicates the 2016 release of the Centers for Disease Control and Prevention’s “Guideline for Prescribing Opioids for Chronic Pain”^10^; and postguideline indicates after the release.

^b^ Patients without commercial insurance are those with Medicare Advantage.

### Prescribing Rates Among Patients Who Are Opioid Naive

**Figure 1.**
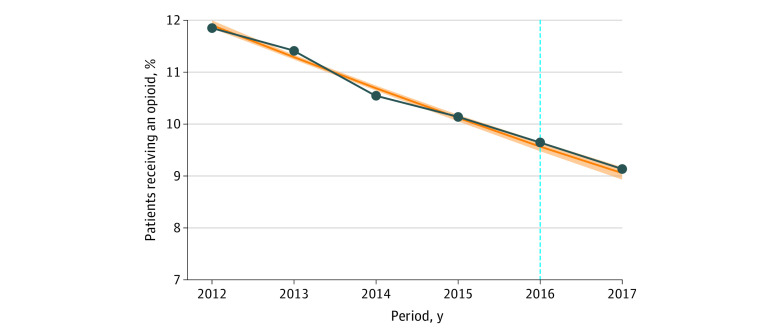
Patients Who Were Opioid Naive and Who Subsequently Received an Opioid Prescription vs Linear Projections Based on Preguideline Trend The vertical blue line indicates the 2016 release of the Centers for Disease Control and Prevention’s “Guideline for Prescribing Opioids for Chronic Pain”^10^; orange line, projected opioid prescriptions based on linear projections from preguideline trend; shading, 95% CI; dark blue line, observed opioid prescriptions.

Opioid prescribing among patients who were opioid naive during the 9-month follow-up period decreased in every cohort compared with the last, dropping from 558 175 patients (11.9%) in preguideline cohort 1 to 532 962 patients (9.1%) in postguideline cohort 2. Prior to the CDC guideline, prescribing during the follow-up period decreased approximately linearly, and the postguideline cohorts continued that trend (Figure 1), although the rates were statistically significantly higher than projected from the preguideline trend. Prescribing rates during follow-up were higher among women and among White patients compared with patients of who were not White (eg, Black, Hispanic, or Asian patients, or those with a missing value for the race/ethnicity variable). Results in the postguideline period were similar, with higher rates than those projected from the preexisting trend (eTable 7 in the Supplement).

### Initial Prescribing Duration

**Figure 2.**
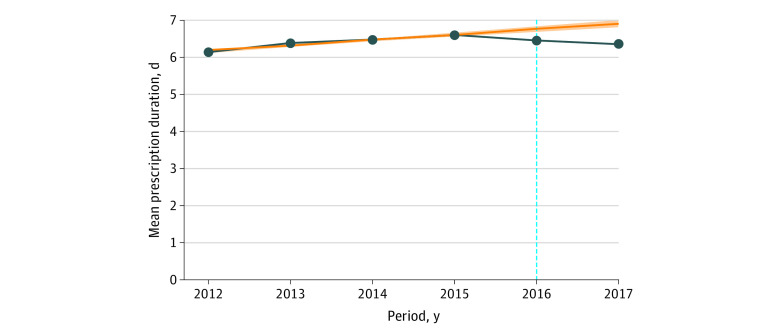
Mean Initial Prescription Duration Among Patients Who Were Opioid Naive Receiving Any Opioid Prescription vs Projections From Preguideline Trend The vertical blue line indicates the 2016 release of the Centers for Disease Control and Prevention’s “Guideline for Prescribing Opioids for Chronic Pain”^10^; orange line, projected opioid prescription duration based on linear projections from preguideline trend; shading, 95% CI; dark blue line, observed opioid prescription duration.

Across cohorts, the duration of the initial prescription was a mean (SD) of 6.4 (7.0) days, and the preguideline means were increasing approximately linearly. That trend was reversed in the postguideline cohorts (Figure 2). Duration projections based on the preexisting secular trend were higher than the observed supply duration in the first (projected: 6.80 [95% CI, 6.80-6.80] days; observed: mean [SD], 6.49 [6.99] days), and second (projected: 6.95 [95% CI, 6.95-6.95] days; observed: mean [SD], 6.38 [7.10] days) postguideline cohorts. Analogous results were seen across demographic subsets, in which the mean days’ supply was lower in the second postguideline group compared with the preexisting trend (eTable 8 in the Supplement). There were differences in initial prescription duration across subsets of patients, with White patients (compared with non-White patients), men (compared with women), and commercially insured patients (compared with Medicare Advantage patients) showing lower mean initial duration (eTable 8 in the Supplement). Further description of the distribution of initial prescription duration is shown in eFigure 3 in the Supplement.

Poisson regression analysis (with overdispersion correction) of initial prescribing duration is shown in Table 2. These results are consistent with the unadjusted outcomes, showing an observed days’ supply in the postguideline periods less than levels projected from a continuation of the preguideline trend. Specifically, mean prescription duration was significant lower in the first (rate ratio, 0.95; 95% CI, 0.95-0.96) and second year (rate ratio, 0.90; 95% CI, 0.90-0.91), after controlling with patient demographics and state fixed effects. Older patients, women, non-White patients, and Medicare Advantage patients had longer initial prescriptions (Table 2).

**Table 2.** Poisson Regression of Initial Opioid Prescription Duration Among Patients Who Were Opioid Naive and Who Subsequently Received an Opioid Prescription and Logistic Regression of Initial Prescription 50 MME Per Day or More

Variable	Initial prescription duration, rate ratio (95% CI)^a^^,^^b^	Initial prescription ≥50 MME/d, odds ratio (95% CI)^b^
Time, y	1.01 (1.01-1.01)	1.00 (0.997-1.00)
Age	1.01 (1.01-1.01)	0.995 (0.99-0.995)
Men^c^	0.96 (0.96-0.97)	1.09 (1.08-1.09)
White race^d^	0.95 (0.95-0.95)	1.24 (1.23-1.24)
Commercial insurance^e^	0.83 (0.82-0.83)	1.12 (1.11-1.13)
First year post-guideline	0.95 (0.95-0.96)	0.97 (0.96-0.98)
Second year post-guideline	0.90 (0.90-0.91)	0.94 (0.93-0.96)

Abbreviation: MME, morphine milligram equivalents.

^a^ Includes correction for overdispensation.

^b^ Includes state-fixed effects.

^c^ The reference group was women.

^d^ The reference group was non-White race/ethnicity, including Asian, Black, Hispanic, and unknown race/ethnicity.

^e^ The reference group was Medicare Advantage insurance.

### Initial Prescribing Dosage

**Figure 3.**
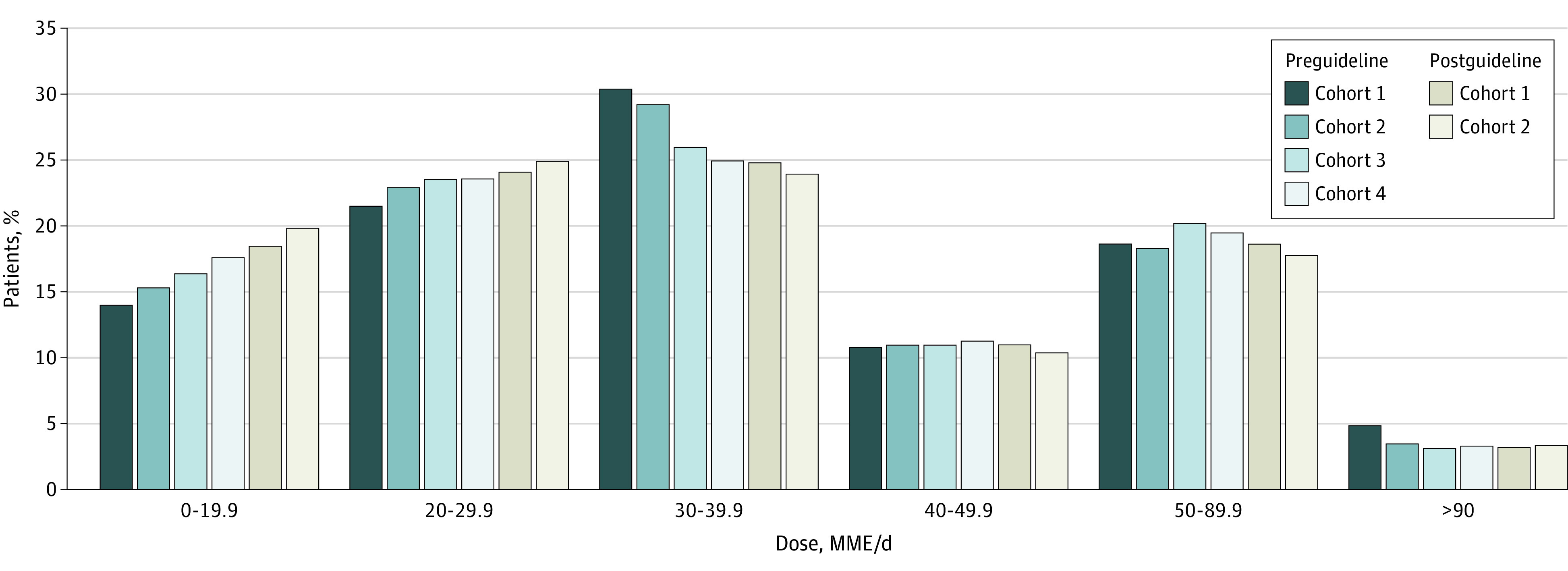
Distribution of Initial Doses of New Opioid Prescriptions in Morphine Milligram Equivalents per Day in Each Year Among Patients Who Were Opioid Naive and Who Subsequently Received Opioids, United States, 2011-2017 Preguideline indicates the 2016 release of the Centers for Disease Control and Prevention’s “Guideline for Prescribing Opioids for Chronic Pain”^10^; postguideline indicates after the release.

Figure 3 shows the frequency distribution of initial doses across cohorts. There were increasing proportions across time of initial dosages that were less than 30 MME/d, and this trend predated the CDC guideline. Additionally, there were decreasing proportions of dosages of 50 to 90 MME/d beginning in preguideline cohort 3, and the proportion of dosages in this category continued to drop through postguideline cohort 2. Compensatory increases were observed in the increased frequency of initial doses in the 0 to 29.9 MME/d range (Figure 3).

eTable 9 in the Supplement shows rates of initial dosages of 50 MME/d or more by cohort. There were reductions in high-dose initial prescribing compared with projections based on the preexisting trend evident by postguideline cohort 2 (projected: 22.5% (95% CI, 22.3%-22.7%); observed: 112 312 patients [21.1%]). Similar reductions were observed in other patient subpopulations, with the exception of Medicare Advantage patients, in whom the observed reductions in initial prescriptions of 50 MME/d or more overshot the preexisting secular trend. Logistic regression analysis of prescriptions of 50 MME/d or more is shown in Table 2. Consistent with the unadjusted analyses, men, White patients, and those with commercial insurance were more likely to be initiated at 50 MME/d or more. Adjusting for state-level effects and for secular time trends, the odds of 50 MME/d or greater initial prescribing were lower in the first (odds ratio, 0.97; 95% CI, 0.96-0.98) and second (odds ratio, 0.94 [95% CI, 0.93-0.96) years after the CDC guideline release compared with projected rates based on preguideline trends.

## Discussion

This cohort study was the first evaluation, to our knowledge, of changes in opioid initiation, dosage, and duration among patients who were opioid naive after the release of the CDC guideline.^15^ Rates of opioid initiation decreased every year from 2012 to 2017, predating the CDC guideline, and our analysis suggests that postguideline reductions in opioid initiation were smaller in magnitude than projected based on the preguideline trend. However, our results suggest the dynamics of initial prescribing duration and the likelihood of being started at 50 MME/d or greater changed after the guideline release. Specifically, an increasing trend in initial prescribing duration was reversed after the CDC guideline was released. In addition, the proportions of patients with initial doses of 50 MME/d or greater were lower following the guideline release than what would be consistent with a continuation of the preexisting trend, with evidence suggesting a corresponding increase in doses starting at less than 30 MME/d.

While prior work, such as a 2018 study by Bohnert et al,^11^ has examined changes in prescription opioid dispensing after the release of the guideline at a population level, our ability to determine how initial dosages changed highlights the value added by this analysis of the treatment received by patients who were opioid naive. The analysis by Bohnert et al^11^ showed that the CDC guideline coincided with acceleration of the decreasing prescription opioid dispensing trajectory. In contrast, our analysis was able to examine whether the treatment received was consistent with guideline language, suggesting greater care in initiating opioid therapy with regard to the starting dose and duration.

Our findings of reduced mean initial days’ supply and reduced initial dosage levels are consistent with clinicians changing their practices in ways recommended by the CDC guideline. Recent work in the context of postoperative pain management^16^ and among individuals receiving long-term opioid therapy^17^ indicates that reductions in the quantity of opioids prescribed are not accompanied by increases in pain intensity, which suggests that these changes may not come at the sacrifice of effective pain management. However, findings from a 2019 study by Fenton et al^12^ suggest that routine clinical practice is changing in ways that are not consistent with guideline language, such as increased rates of rapid tapering or abrupt discontinuation. More work is needed to clarify changes in clinical practice and patient outcomes coinciding with the CDC guideline release.

Reductions in initial opioid prescribing dosage and duration may correspond to reductions in several other risks. Longer initial days’ supply is associated with higher probability of continued opioid use 1 to 3 years later,^18^ and there is evidence this is the strongest risk factor for continued use.^19,20^ In addition, higher initial doses, particularly at levels of 45 MME/d or more, are associated with increased probability of long-term opioid use.^20^ Given that long-term opioid use is associated with several risks,^21^ including misuse and substance use disorder,^22,23,24,25^ opioid overdose,^26,27^ injuries,^28,29^ and cardiovascular disease,^30^ changes in clinical practice may correspond to reductions in several other opioid-related harms. Future work should examine whether there were corresponding changes in untreated pain or in the uptake of treatments suggested by the CDC guideline,^15^ such as nonpharmacological treatments (eg, cognitive behavioral therapy), and nonopioid pain medications (eg, non-steroidal anti-inflammatory drugs, acetaminophen).

Our analysis suggests that changes in initial days’ supply and initial dosage after the CDC guideline release accumulated with time, because the changes were greater in the 2016 to 2017 cohort than the 2015 to 2016 cohort. This accelerating change is consistent with related work, eg, an analysis by Lin et al^31^ found that after the implementation of the Opioid Safety Initiative designed by the Department of Veteran Affairs, the departure of rates of high-dose prescribing from the preexisting secular trends increased over time. Our findings are consistent with there being an acclimation period required before the outcomes associated with the guideline were realized. This acclimation period is likely ongoing, particularly in terms of the shifting culture around opioid prescribing, of which the CDC guideline was part, contributing to broader changes in insurer practices. For example, misapplication of the CDC guideline has been reported for patients with pain associated with cancer, surgical procedures, or acute sickle cell crises—all of whom are patient populations for which the CDC guideline does not apply.^32,33^ This suggests a more robust focus on implementation of evidence-based guidelines and emphasizing support for guideline-concordant care as methods for hastening the benefits of such guidelines.

The primary subpopulation-specific differences we found in the changes after the release of the CDC guideline were between commercially insured and Medicare Advantage patients. Specifically, reductions in high (ie, ≥50 MME/d) initial dosages were seen across subpopulations compared with the preguideline trend, except among Medicare Advantage patients. Future research is needed to explore reasons for differences in postguideline prescribing among Medicare Advantage patients, which may yield strategies for optimized guideline implementation.

Other secular changes in the opioid policy environment, at least partially, coincided temporally with the CDC guideline release. For example, the 21st Century Cures Act,^34^ enacted in December 2016, provided states with financial resources to combat the opioid crisis, including supporting efforts to address opioid prescribing. Similarly, by April 2017, prescription drug monitoring programs, which are secure, online, state-based databases that contain information about controlled substance prescriptions written by a clinician and dispensed by a pharmacist, were implemented across the US. The central aim of a prescription drug monitoring program is to reduce the misuse and diversion of controlled substances, like prescription opioids, and widespread use of these programs could impact prescribing, such as limiting the degree to which patients can obtain multiple overlapping prescriptions. Thus, it is not possible to fully differentiate the outcomes associated with the CDC guideline from those of other coinciding countermeasures. Yet, the existence of other countermeasures may reflect a shift in the response to the opioid crisis—a shift of which the CDC guideline is part and may have helped to catalyze, making evaluations of changes around that time important, despite limitations to causal attributions. Implementation of the CDC guideline included assistance to states, insurers, health systems, and other policy makers to design interventions^35^ informed by the specific recommendations within the CDC guideline. Thus, the outcomes of some of these concurrent programs may be considered as part of the total outcome (including indirect outcomes) associated with the CDC guideline on prescribing.

### Limitations

There are some limitations of this study. First, our inclusion criteria, which did not include uninsured or Medicaid patients and required continuous enrollment to determine opioid naive status, may select for higher socioeconomic status. More generally, claims data are susceptible to unmeasured confounding, particularly through changes in the study population over time. Future work is required to determine if our findings generalize to the broader US population. Second, these data cannot capture self-paid prescriptions, which may result in underestimates of prescribing. However, prior data suggest that self-payment for opioid prescriptions was becoming increasingly less common.^36^ Third, these data did not permit the measurement of potential confounders, such as pain intensity, pain-related functioning, and quality of life. Fourth, there are differences in how institutions codify and provide guidance on how to implement to the CDC guideline^33^ that cannot be fully accounted for in this analysis. Fifth, the increased frequency of missing data in the race/ethnicity variable derived from Clinformatics limited the ability to easily interpret demographic differences in prescribing.

## Conclusions

This cohort study found that prescribing for initial opioid use changed after the release of the CDC guideline, suggesting that evidence-based guidelines from trusted sources may be associated with clinician behavior. Further improvements in opioid prescribing practices may be achieved by lessening unintended consequences of the CDC guideline, such as rapid tapering,^12^ that may result from misapplication of the guideline language.^32,33^ Effective implementation would include assessment of benefits and risks, patient education, and risk mitigation. Additionally, expanding access to nonopioid therapy, including nonopioid pain medications and nonpharmacologic therapies, coupled with technological innovations, such as electronic health record–based dashboards for clinicians to track pain treatment practices and outcomes for patients who are opioid naive, could help pain management. These and other strategies to optimize guideline-concordant care have potential to improve pain management and reduce opioid-related harms.

## Notes

Supplement. **eFigure 1.** Schematic Diagram of the Time Windows for Each Preguideline and Postguideline Cohort **eFigure 2.** Proportion of All Beneficiaries Who Disenrolled From Coverage During Each Month **eFigure 3.** Boxplot of Initial Prescription Durations Among Patients Who Were Opioid Naive in Each Cohort **eTable 1.** Cancer and Palliative Care Diagnosis Codes Used for Exclusion Criteria **eTable 2.** Age Distribution by Cohort Among Commercially Insured, Medicare Advantage Patients, and the Total Cohort **eTable 3.** Race/Ethnicity Distribution by Cohort Among Commercially Insured, Medicare Advantage Patients, and the Total Cohort **eTable 4.** Region Distribution by Cohort and Stratified by Insurer Type **eTable 5.** Sex Distribution by Cohort Among Commercially Insured, Medicare Advantage Patients, and the Total Cohort **eTable 6.** NHIS-Based Estimates of Commercially Insured Patient Demographics and Medicare Plus Insured Patient Demographics **eTable 7.** Percentage of Enrollees Who Received a New Opioid Prescription During the Follow-up Period in Each Cohort **eTable 8.** Mean Duration of Initial Opioid Prescription Among Patients Who Were Opioid Naive and Who Subsequently Received an Opioid Prescription by Cohort and Demographic; United States, 2011-2017 **eTable 9.** Percentage of Patients Who Were Opioid Naive and Who Subsequently Received an Opioid Prescription at 50 Morphine Milligram Equivalents Per Day or More
