# Supplementary material for: Changes in Initial Opioid Prescribing Practices After the 2016 Release of the CDC Guideline for Prescribing Opioids for Chronic Pain
Source: JAMA Netw Open. 2021 Jul 13;4(7):e2116860. doi: 10.1001/jamanetworkopen.2021.16860 (PMC8278262; doi:10.1001/jamanetworkopen.2021.16860)
Supplement: Supplement. — eFigure 1. Schematic Diagram of the Time Windows for Each Preguideline and Postguideline Cohort eFigure 2. Proportion of All Beneficiaries Who Disenrolled From Coverage During Each Month eFigure 3. Boxplot of Initial Prescription Durations Among Patients Who Were Opioid Naive in Each Cohort eTable 1. Cancer and Palliative Care Diagnosis Codes Used for Exclusion Criteria eTable 2. Age Distribution by Cohort Among Commercially Insured, Medicare Advantage Patients, and the Total Cohort eTable 3. Race/Ethnicity Distribution by Cohort Among Commercially Insured, Medicare Advantage Patients, and the Total Cohort eTable 4. Region Distribution by Cohort and Stratified by Insurer Type eTable 5. Sex Distribution by Cohort Among Commercially Insured, Medicare Advantage Patients, and the Total Cohort eTable 6. NHIS-Based Estimates of Commercially Insured Patient Demographics and Medicare Plus Insured Patient Demographics eTable 7. Percentage of Enrollees Who Received a New Opioid Prescription During the Follow-up Period in Each Cohort eTable 8. Mean Duration of Initial Opioid Prescription Among Patients Who Were Opioid Naive and Who Subsequently Received an Opioid Prescription by Cohort and Demographic; United States, 2011-2017 eTable 9. Percentage of Patients Who Were Opioid Naive and Who Subsequently Received an Opioid Prescription at 50 Morphine Milligram Equivalents Per Day or More [file jamanetwopen-e2116860-s001.pdf]

Supplemental Online Content

Goldstick JE, Guy GP, Losby JL, Baldwin G, Myers M, Bohnert ASB. Changes in initial opioid prescribing practices after the 2016 release of the CDC Guideline for Prescribing Opioids for Chronic Pain. *JAMA Netw Open*. 2021;4(7):e2116860. doi:10.1001/jamanetworkopen.2021.16860

eFigure 1. Schematic Diagram of the Time Windows for Each Preguideline and Postguideline Cohort

eFigure 2. Proportion of All Beneficiaries Who Disenrolled From Coverage During Each Month

eFigure 3. Boxplot of Initial Prescription Durations Among Patients Who Were Opioid Naive in Each Cohort

eTable 1. Cancer and Palliative Care Diagnosis Codes Used for Exclusion Criteria

eTable 2. Age Distribution by Cohort Among Commercially Insured, Medicare Advantage Patients, and the Total Cohort

eTable 3. Race/Ethnicity Distribution by Cohort Among Commercially Insured, Medicare Advantage Patients, and the Total Cohort

eTable 4. Region Distribution by Cohort and Stratified by Insurer Type

eTable 5. Sex Distribution by Cohort Among Commercially Insured, Medicare Advantage Patients, and the Total Cohort

eTable 6. NHIS-Based Estimates of Commercially Insured Patient Demographics and Medicare Plus Insured Patient Demographics

eTable 7. Percentage of Enrollees Who Received a New Opioid Prescription During the Follow-up Period in Each Cohort

eTable 8. Mean Duration of Initial Opioid Prescription Among Patients Who Were Opioid Naive and Who Subsequently Received an Opioid Prescription by Cohort and Demographic; United States, 2011-2017

eTable 9. Percentage of Patients Who Were Opioid Naive and Who Subsequently Received an Opioid Prescription at 50 Morphine Milligram Equivalents Per Day or More

This supplemental material has been provided by the authors to give readers additional information about their work.

© 2021 Goldstick JE et al. *JAMA Network Open.*

eFigure 1. Schematic Diagram of the Time Windows for Each Preguideline and Postguideline Cohort

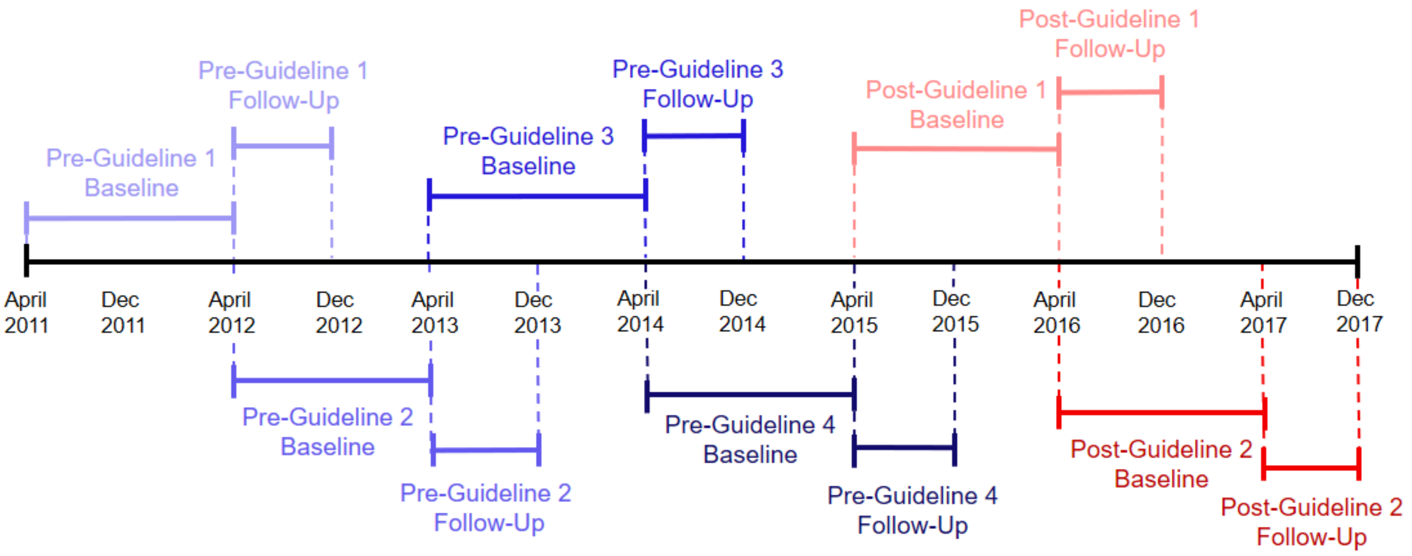

Note: To qualify for each cohort individuals were required to: 1) be continuously enrolled for the entire 21-month period; 2) have 0 total Morphine Milligram Equivalents (MMEs) during their baseline period; and 3) Not having any diagnoses for cancer, palliative care, or end-of-life care at any time from 2011-2018.

© 2021 Goldstick JE et al. *JAMA Network Open.*

eFigure 2. Proportion of All Beneficiaries Who Disenrolled From Coverage During Each Month

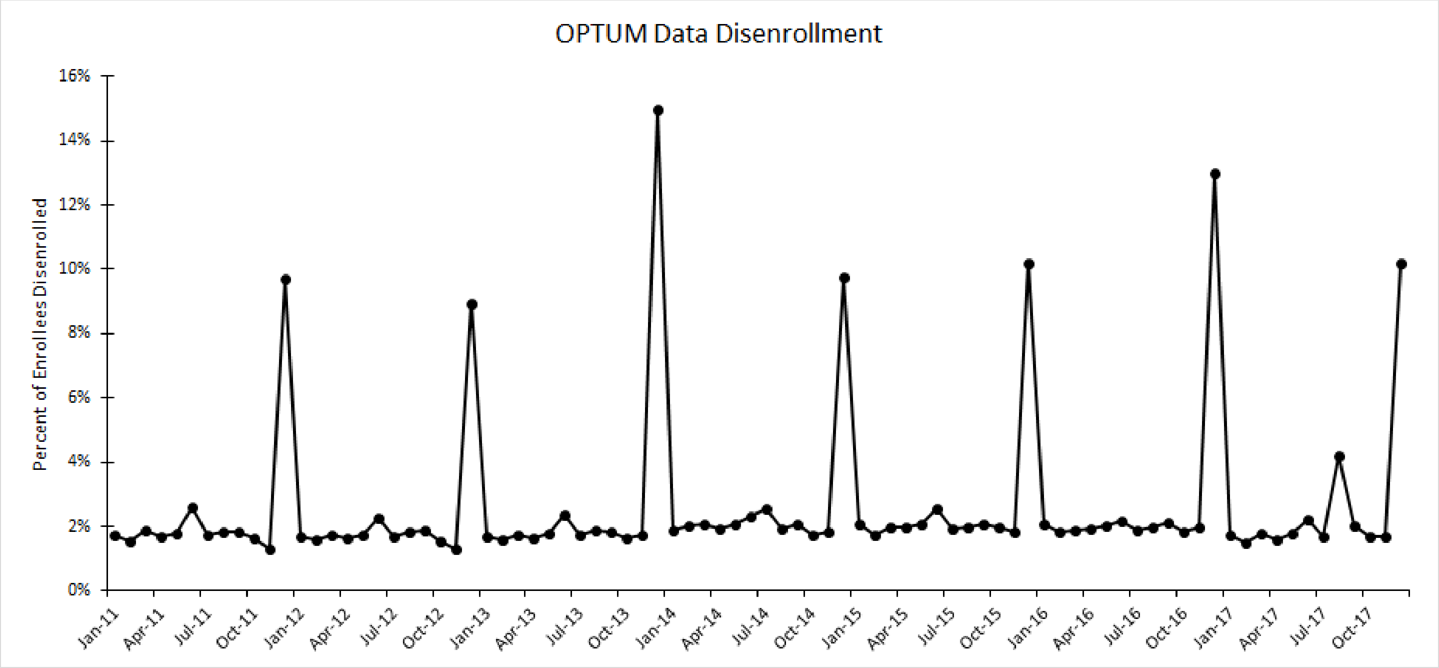

© 2021 Goldstick JE et al. *JAMA Network Open.*

eFigure 3. Boxplot of Initial Prescription Durations Among Patients Who Were Opioid Naive in Each Cohort

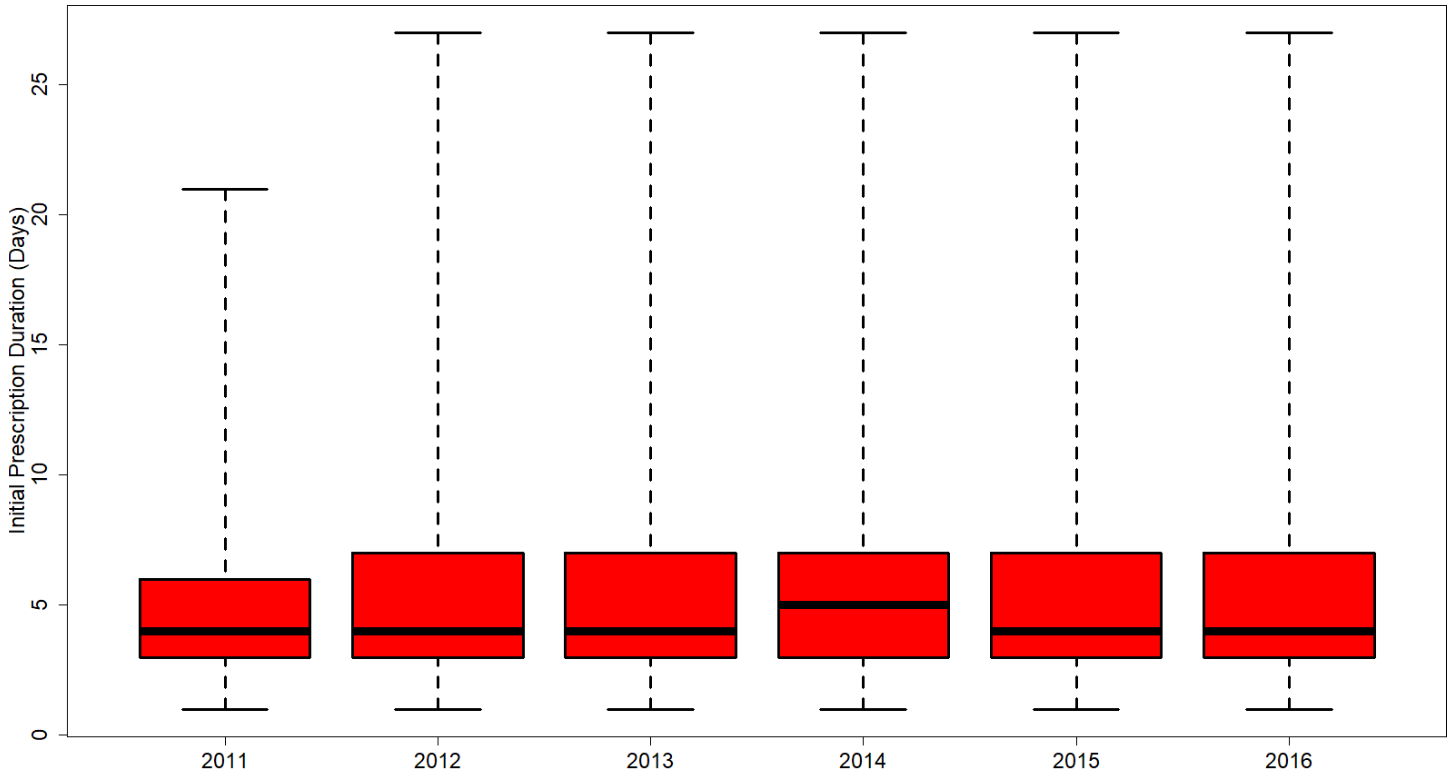

Note: Whiskers extend up to five times the interquartile range above the 75th percentile

© 2021 Goldstick JE et al. *JAMA Network Open.*

eTable 1. Cancer and Palliative Care Diagnosis Codes Used for Exclusion Criteria

**Table X** ICD-9 CM, ICD-10 CM, Cancer/Palliative Care Code Exclusions		
	**ICD-9**	**ICD-10**
Cancer (Malignant Neoplasm) Diagnosis*	140.X–208.X, 209.0–209.3, V10.X	C00X–C97X
Palliative Care	V66.7	Z51.5
*Excluding benign neoplasms or neoplasms of uncertain behavior		

© 2021 Goldstick JE et al. *JAMA Network Open.*

eTable 2. Age Distribution by Cohort Among Commercially Insured, Medicare Advantage Patients, and the Total Cohort

Commercial				
Year	Age 18-34	Age 35-54	Age 55-74	Age 75+
Pre-Guideline Cohort 1	1,171,553	1,737,007	713,343	42,275
	(32.0%)	(47.4%)	(19.5%)	(1.2%)
Pre-Guideline Cohort 2	1,193,784	1,721,370	723,113	30,174
	(32.5%)	(46.9%)	(19.7%)	(0.8%)
Pre-Guideline Cohort 3	1,104,662	1,534,174	632,629	26,162
	(33.5%)	(46.5%)	(19.2%)	(0.8%)
Pre-Guideline Cohort 4	1,182,535	1605676	670,712	25,336
	(33.9%)	(46.1%)	(19.2%)	(0.7%)
Post-Guideline Cohort 1	1,233,130	1,675,721	742,793	26,060
	(33.5%)	(45.6%)	(20.2%)	(0.7%)
Post-Guideline Cohort 2	1,328,032	1,776,120	795,678	29,371
	(33.8%)	(45.2%)	(20.3%)	(0.7%)
**Medicare**				
**Year**	**Age 18-34**	**Age 35-54**	**Age 55-74**	**Age 75+**
Pre-Guideline Cohort 1	3,185	29,354	602,388	406,988
	(0.3%)	(2.8%)	(57.8%)	(39.1%)
Pre-Guideline Cohort 2	3,754	33,285	699,762	464,570
	(0.3%)	(2.8%)	(58.2%)	(38.7%)
Pre-Guideline Cohort 3	3,998	33,736	684,874	455,483
	(0.3%)	(2.9%)	(58.1%)	(38.7%)
Pre-Guideline Cohort 4	4,477	34,275	758,808	517,991
	(0.3%)	(2.6%)	(57.7%)	(39.4%)
Post-Guideline Cohort 1	5,130	37,450	891,168	623,506
	(0.3%)	(2.4%)	(57.2%)	(40.0%)
Post-Guideline Cohort 2	6,842	49,002	1,097,259	751,784
	(0.4%)	(2.6%)	(57.6%)	(39.5%)
**Total**				
**Year**	**Age 18-34**	**Age 35-54**	**Age 55-74**	**Age 75+**
Pre-Guideline Cohort 1	1,174,738	1,766,361	1,315,731	449,263
	(25.0%)	(37.5%)	(28.0%)	(9.5%)
Pre-Guideline Cohort 2	1,197,538	1,754,655	1,422,875	494,744
	(24.6%)	(36.0%)	(29.2%)	(10.2%)
Pre-Guideline Cohort 3	1,108,660	1,567,910	1,317,503	481,645
	(24.8%)	(35.0%)	(29.4%)	(10.8%)
Pre-Guideline Cohort 4	1,187,012	1,639,951	1,429,520	543,327
	(24.7%)	(34.2%)	(29.8%)	(11.3%)
Post-Guideline Cohort 1	1,238,260	1,713,171	1,633,961	649,566
	(23.7%)	(32.7%)	(31.2%)	(12.4%)
Post-Guideline Cohort 2	1,334,874	1,825,122	1,892,937	781,155
	(22.9%)	(31.3%)	(32.4%)	(13.4%)

© 2021 Goldstick JE et al. *JAMA Network Open.*

eTable 3. Race/Ethnicity Distribution by Cohort Among Commercially Insured, Medicare Advantage Patients, and the Total Cohort

Commercial					
Year	Unknown	Asian	Black	Hispanic	White
Pre-Guideline Cohort 1	243,323	199,454	345,489	396,516	2,479,396
	(6.6%)	(5.4%)	(9.4%)	(10.8%)	(67.7%)
Pre-Guideline Cohort 2	241,506	208,108	340,178	401,333	2,477,316
	(6.6%)	(5.7%)	(9.3%)	(10.9%)	(67.5%)
Pre-Guideline Cohort 3	219,272	196,918	271,081	395,276	2,215,080
	(6.6%)	(6.0%)	(8.2%)	(12.0%)	(67.2%)
Pre-Guideline Cohort 4	238,780	207,589	291,130	431,157	2,315,603
	(6.9%)	(6.0%)	(8.4%)	(12.4%)	(66.5%)
Post-Guideline Cohort 1	240,044	221,202	308,458	469,137	2,438,863
	(6.5%)	(6.0%)	(8.4%)	(12.8%)	(66.3%)
Post-Guideline Cohort 2	413,438	224,491	310,124	488,546	2,492,602
	(10.5%)	(5.7%)	(7.9%)	(12.4%)	(63.4%)
**Medicare**					
Year	Unknown	Asian	Black	Hispanic	White
Pre-Guideline Cohort 1	179,412	31,210	87,398	93,530	650,365
	(17.2%)	(3.0%)	(8.4%)	(9.0%)	(62.4%)
Pre-Guideline Cohort 2	183,748	50,485	105,652	111,526	749,960
	(15.3%)	(4.2%)	(8.8%)	(9.3%)	(62.4%)
Pre-Guideline Cohort 3	180,123	53,078	95,674	116,198	733,018
	(15.3%)	(4.5%)	(8.1%)	(9.9%)	(62.2%)
Pre-Guideline Cohort 4	196,927	57,703	107,113	144,247	809,561
	(15.0%)	(4.4%)	(8.1%)	(11.0%)	(61.5%)
Post-Guideline Cohort 1	237,734	64,285	133,992	159,567	961,676
	(15.3%)	(4.1%)	(8.6%)	(10.2%)	(61.8%)
Post-Guideline Cohort 2	289,295	69,534	191,135	187,376	1,167,547
	(15.2%)	(3.7%)	(10.0%)	(9.8%)	(61.3%)
**Total**					
Year	Unknown	Asian	Black	Hispanic	White
Pre-Guideline Cohort 1	422,735	230,664	432,887	490,046	3,129,761
	(9.0%)	(4.9%)	(9.2%)	(10.4%)	(66.5%)
Pre-Guideline Cohort 2	425,254	258,593	445,830	512,859	3,227,276
	(8.7%)	(5.3%)	(9.2%)	(10.5%)	(66.3%)
Pre-Guideline Cohort 3	399,395	249,996	366,755	511,474	2,948,098
	(8.9%)	(5.6%)	(8.2%)	(11.4%)	(65.9%)
Pre-Guideline Cohort 4	435,707	265,292	398,243	575,404	3,125,164
	(9.1%)	(5.5%)	(8.3%)	(12.0%)	(65.1%)
Post-Guideline Cohort 1	477,778	285,487	442,450	628,704	3,400,539
	(9.1%)	(5.5%)	(8.5%)	(12.0%)	(65.0%)
Post-Guideline Cohort 2	702,733	294,025	501,259	675,922	3,660,149
	(12.0%)	(5.0%)	(8.6%)	(11.6%)	(62.7%)

© 2021 Goldstick JE et al. *JAMA Network Open.*

eTable 4. Region Distribution by Cohort and Stratified by Insurer Type

Commercial						
**Year**	**West**	**Midwest**	**South**	**Northeast**	**Pacific**	**Unknown**
Pre-Guideline Cohort 1	730,598	944,594	1,590,444	380,466	2,481	15,595
	(19.9%)	(25.8%)	(43.4%)	(10.4%)	(0.1%)	(0.4%)
Pre-Guideline Cohort 2	750,380	964,323	1,555,238	368,040	2,510	27,950
	(20.5%)	(26.3%)	(42.4%)	(10.0%)	(0.1%)	(0.8%)
Pre-Guideline Cohort 3	719,034	911,726	1,297,445	330,208	2,223	36,991
	(21.8%)	(27.6%)	(39.3%)	(10.0%)	(0.1%)	(1.1%)
Pre-Guideline Cohort 4	748,507	958,751	1,374,387	348,808	2,666	51,140
	(21.5%)	(27.5%)	(39.4%)	(10.0%)	(0.1%)	(1.5%)
Post-Guideline Cohort 1	784,533	1,008,291	1,460,199	377,425	2,831	44,425
	(21.3%)	(27.4%)	(39.7%)	(10.3%)	(0.1%)	(1.2%)
Post-Guideline Cohort 2	838,609	1,039,149	1,495,979	379,416	2,852	173,196
	(21.3%)	(26.4%)	(38.1%)	(9.7%)	(0.1%)	(4.4%)
**Medicare**						
**Year**	**West**	**Midwest**	**South**	**Northeast**	**Pacific**	**Unknown**
Pre-Guideline Cohort 1	375,379	215,979	338,290	85,827	9,402	17,038
	(36.0%)	(20.7%)	(32.5%)	(8.2%)	(0.9%)	(1.6%)
Pre-Guideline Cohort 2	383,575	247,390	373,364	170,918	9,779	16,345
	(31.9%)	(20.6%)	(31.1%)	(14.2%)	(0.8%)	(1.4%)
Pre-Guideline Cohort 3	382,503	262,451	324,018	180,257	8,451	20,411
	(32.5%)	(22.3%)	(27.5%)	(15.3%)	(0.7%)	(1.7%)
Pre-Guideline Cohort 4	397,713	298,967	398,725	206,748	8,966	4,432
	(30.2%)	(22.7%)	(30.3%)	(15.7%)	(0.7%)	(0.3%)
Post-Guideline Cohort 1	431,229	343,839	524,222	241,461	11,634	4,869
	(27.7%)	(22.1%)	(33.7%)	(15.5%)	(0.7%)	(0.3%)
Post-Guideline Cohort 2	483,479	431,406	693,799	284,398	6,461	5,344
	(25.4%)	(22.6%)	(36.4%)	(14.9%)	(0.3%)	(0.3%)
**Total**						
**Year**	**West**	**Midwest**	**South**	**Northeast**	**Pacific**	**Unknown**
Pre-Guideline Cohort 1	1,105,977	1,160,573	1,928,734	466,293	11,883	32,633
	(23.5%)	(24.7%)	(41.0%)	(9.9%)	(0.3%)	(0.7%)
Pre-Guideline Cohort 2	1,133,955	1,211,713	1,928,602	538,958	12,289	44,295
	(23.3%)	(24.9%)	(39.6%)	(11.1%)	(0.3%)	(0.9%)
Pre-Guideline Cohort 3	1,101,537	1,174,177	1,621,463	510,465	10,674	57,402
	(24.6%)	(26.2%)	(36.2%)	(11.4%)	(0.2%)	(1.3%)
Pre-Guideline Cohort 4	1,146,220	1,257,718	1,773,112	555,556	11,632	55,572
	(23.9%)	(26.2%)	(36.9%)	(11.6%)	(0.2%)	(1.2%)
Post-Guideline Cohort 1	1,215,762	1,352,130	1,984,421	618,886	14,465	49,294
	(23.2%)	(25.8%)	(37.9%)	(11.8%)	(0.3%)	(0.9%)
Post-Guideline Cohort 2	1,322,088	1,470,555	2,189,778	663,814	9,313	178,540
	(22.7%)	(25.2%)	(37.5%)	(11.4%)	(0.2%)	(3.1%)

Regional categories based on census categories West, Midwest, Northeast, and South (https://www2.census.gov/geo/pdfs/maps-data/maps/reference/us_regdiv.pdf). Additionally, added “Pacific” to refer to Puerto Rico, Hawaii, and Alaska.

© 2021 Goldstick JE et al. *JAMA Network Open.*

eTable 5. Sex Distribution by Cohort Among Commercially Insured, Medicare Advantage Patients, and the Total Cohort

Commercial			
Year	Male	Female	Unknown
Pre-Guideline Cohort 1	1,876,731	1,786,748	699
	(51.2%)	(48.8%)	(0.0%)
Pre-Guideline Cohort 2	1,888,038	1,779,763	640
	(51.5%)	(48.5%)	(0.0%)
Pre-Guideline Cohort 3	1,727,385	1,569,691	551
	(52.4%)	(47.6%)	(0.0%)
Pre-Guideline Cohort 4	1,825,382	1,658,428	449
	(52.4%)	(47.6%)	(0.0%)
Post-Guideline Cohort 1	1,922,439	1,754,910	355
	(52.3%)	(47.7%)	(0.0%)
Post-Guideline Cohort 2	2,071,439	1,857,466	296
	(52.7%)	(47.3%)	(0.0%)
**Medicare**			
**Year**	**Male**	**Female**	**Unknown**
Pre-Guideline Cohort 1	437,213	604,702	0
	(42.0%)	(58.0%)	(0.0%)
Pre-Guideline Cohort 2	508,666	692,705	0
	(42.3%)	(57.7%)	(0.0%)
Pre-Guideline Cohort 3	503,385	674,706	0
	(42.7%)	(57.3%)	(0.0%)
Pre-Guideline Cohort 4	551,168	764,383	0
	(41.9%)	(58.1%)	(0.0%)
Post-Guideline Cohort 1	643,805	913,449	0
	(41.3%)	(58.7%)	(0.0%)
Post-Guideline Cohort 2	788,664	1,116,223	0
	(41.4%)	(58.6%)	(0.0%)
**Total**			
**Year**	**Male**	**Female**	**Unknown**
Pre-Guideline Cohort 1	2,313,944	2,391,450	699
	(49.2%)	(50.8%)	(0.0%)
Pre-Guideline Cohort 2	2,396,704	2,472,468	640
	(49.2%)	(50.8%)	(0.0%)
Pre-Guideline Cohort 3	2,230,770	2,244,397	551
	(49.8%)	(50.1%)	(0.0%)
Post-Guideline Cohort 4	2,376,550	2,422,811	449
	(49.5%)	(50.5%)	(0.0%)
Post-Guideline Cohort 1	2,566,244	2,668,359	355
	(49.0%)	(51.0%)	(0.0%)
Pre-Guideline Cohort 2	2,860,103	2,973,689	296
	(49.0%)	(51.0%)	(0.0%)

© 2021 Goldstick JE et al. *JAMA Network Open.*

eTable 6. NHIS-Based Estimates of Commercially Insured Patient Demographics and Medicare Plus Insured Patient Demographics

Among All Commercially Insured				
Year	Age (mean; 95% CI)	Gender (% male, 95% CI)	Race (%White, 95% CI)	Race(%Black, 95% CI)
2012	38.24 (38.06-38.41)	49.0 (48.6-49.4)	83.4 (82.7-84.0)	9.7 (9.1-10.2)
2013	38.29 (38.11-38.46)	49.2 (48.8-49.5)	83.0 (82.3-83.7)	9.8 (9.2-10.3)
2014	38.39 (38.23-38.56)	49.3 (49.0-49.7)	82.7 (82.0-83.3)	9.8 (9.2-10.3)
2015	38.19 (38.02-38.36)	49.1 (48.8-49.5)	81.9 (81.2-82.6)	10.2 (9.7-10.8)
2016	38.38 (38.21-38.55)	49.4 (49.0-49.8)	81.3 (80.2-82.5)	10.4 (9.6-11.3)
2017	38.37 (38.18-38.56)	49.3 (48.9-49.7)	81.1 (79.7-82.4)	10.4 (9.5-11.4)
**Among those Enrolled in Medicare Plus Choice**				
Year	Age (mean; 95% CI)	Gender(% male; 95% CI)	Race (%White; 95% CI)	Race(%Black; 95% CI)
2012	72.21 (71.88-72.54)	43.1 (41.3-44.9)	86.5 (84.6-88.2)	9.4 (8.0-11.1)
2013	71.52 (71.17-71.86)	42.7 (41.1-44.2)	86.6 (84.9-88.1)	8.8 (7.6-10.1)
2014	72.25 (71.93-72.56)	43.9 (42.3-45.6)	86.8 (85.2-88.3)	8.7 (7.5-10.1)
2015	72.11 (71.82-72.40)	44.4 (42.9-45.9)	84.9 (83.1-86.6)	10.1 (8.8-11.6)
2016	71.88 (71.59-72.17)	43.7 (42.3-45.2)	84.8 (82.8-86.6)	10 (8.7-11.6)
2017	71.9 (71.61-72.20)	44.1 (42.7-45.6)	84.2 (82.1-86.1)	10.1 (8.5-11.9)

Note: For comparison with the cohort composition, 2012, 2013, 2014, 2015, 2016, 2017 are most closely aligned temporally with Pre-Guideline Cohort 1, Pre-Guideline Cohort 2, Pre-Guideline Cohort 3, Pre-Guideline Cohort 4, Post-Guideline Cohort 1, and Post-Guideline Cohort 2, respectively.

© 2021 Goldstick JE et al. *JAMA Network Open.*

eTable 7. Percentage of Enrollees Who Received a New Opioid Prescription During the Follow-up Period in Each Cohort

	Total	Female	Male	White	Non-white	Commercial	Medicare Advantage
Pre-Guideline 1	558,175 (11.9%)	310,650 (13.0%)	247,425 (10.7%)	385,855 (12.3%)	134,612 (11.7%)	441,490 (12.0%)	116,685 (11.1%)
Pre-Guideline 2	556,234 (11.4%)	309,423 (12.5%)	246,732 (10.3%)	383,397 (11.9%)	136,385 (11.2%)	421,652 (11.4%)	134,582 (11.2%)
Pre-Guideline 3	472,511 (10.6%)	259,200 (11.5%)	213,251 (9.6%)	325,781 (11.1%)	114,895 (10.2%)	348,682 (10.5%)	123,829 (10.5%)
Pre-Guideline 4	487,131 (10.1%)	269,788 (11.1%)	217,277 (9.1%)	327,349 (10.5%)	120,133 (9.7%)	345,900 (9.8%)	141,231 (10.7%)
Post-Guideline 1	505,155 (9.6%)	280,818 (10.5%)	224,310 (8.7%)	338,484 (10.0%)	127,004 (9.4%)	352,350 (9.5%)	152,805 (9.8%)
Post-Guideline 2	532,962 (9.1%)	297,391 (10.0%)	235,554 (8.2%)	349,555 (9.6%)	134,358 (9.1%)	343,514 (8.7%)	189,448 (9.9%)
(actual-predicted)	**+0.1%**	**+0.1%**	**+0.1%**	**+0.2%**	**+0.7%**	**+0.1%**	**-0.1%**

Note: The final row shows the difference between the observed level in the Post-Guideline group 2, and what was projected based on a linear extrapolation of the pre-guideline trend.

Note 2: Those with missing values for race were excluded from the “white” and “non-white” groups, but were not excluded from any other columns

© 2021 Goldstick JE et al. *JAMA Network Open.*

eTable 8. Mean Duration of Initial Opioid Prescription Among Patients Who Were Opioid Naive and Who Subsequently Received an Opioid Prescription by Cohort and Demographic; United States, 2011-2017

	Overall	Female	Male	White	Non-white	Commercial Insurance	Medicare Advantage
Pre-Guideline 1	6.17	6.31	6.00	6.01	6.54	5.50	8.73
Pre-Guideline 2	6.40	6.57	6.19	6.22	6.79	5.61	8.87
Pre-Guideline 3	6.49	6.65	6.30	6.29	6.93	5.62	8.93
Pre-Guideline 4	6.64	6.84	6.39	6.35	7.04	5.62	9.14
Post-Guideline 1	6.49	6.70	6.24	6.25	6.93	5.52	8.72
Post-Guideline 2	6.38	6.61	6.09	6.11	6.89	5.22	8.49
(actual-pred)	**-0.57**	**-0.56**	**-0.59**	**-0.50**	**-0.51**	**-0.51**	**-0.88**
% change (difference/predicted)	**-8.2%**	**-7.8%**	**-8.8%**	**-7.5%**	**-6.9%**	**-8.8%**	**-9.4%**

Note: The final row shows the difference between the observed level in the Post-Guideline group 2, and what was projected based on a linear extrapolation of the pre-guideline trend. The % reduction (diff/pred) entry shows the percent change in the actual level, relative to the projection.

Note 2: Those with missing values for race were excluded from the “Non-white” group, but were not excluded from any other columns.

© 2021 Goldstick JE et al. *JAMA Network Open.*

eTable 9. Percentage of Patients Who Were Opioid Naive and Who Subsequently Received an Opioid Prescription at 50 Morphine Milligram Equivalents Per Day or More

	Total	Female	Male	White	Non-white	Commercial	Medicare Advantage
Pre-Guideline 1	130,883 (23.4%)	71,046 (22.9%)	59,819 (24.2%)	95,532 (24.8%)	26,672 (19.8%)	103,160 (23.4%)	27,723 (23.8%)
Pre-Guideline 2	120,861 (21.7%)	65,172 (21.1%)	55,675 (22.6%)	88,488 (23.1%)	24,395 (17.9%)	94,390 (22.4%)	26,471 (19.7%)
Pre-Guideline 3	110.017 (23.3%)	58,045 (22.4%)	51,959 (24.4%)	81,103 (24.9%)	21,661 (18.9%)	86,920 (24.9%)	23,097 (18.7%)
Pre-Guideline 4	110,734 (22.7%)	59,049 (21.9%)	51,672 (23.8%)	80,912 (24.7%)	21,375 (17.8%)	84,359 (24.4%)	26,375 (18.7%)
Post-Guideline 1	110,088 (21.8%)	58,693 (20.9%)	51,390 (22.9%)	80,598 (23.8%)	21,172 (16.7%)	82,703 (23.5%)	27,385 (17.9%)
Post-Guideline 2	112,312 (21.1%)	60,116 (20.2%)	52,189 (22.2%)	80,813 (23.1%)	21,591 (16.1%)	79,278 (23.1%)	33,034 (17.4%)
(actual-pred)	**-1.5%**	**-1.2%**	**-1.7%**	**-1.8%**	**-0.7%**	**-2.6%**	**+2.2%**

Note: The final row shows the difference between the observed level in the Post-Guideline group 2, and what was projected based on a linear extrapolation of the pre-guideline trend.

Note 2: Those with missing values for race were excluded from the “Non-white” group, but were not excluded from any other columns.

© 2021 Goldstick JE et al. *JAMA Network Open.*
